# Mouse SAMHD1 Has Antiretroviral Activity and Suppresses a Spontaneous Cell-Intrinsic Antiviral Response

**DOI:** 10.1016/j.celrep.2013.07.037

**Published:** 2013-08-22

**Authors:** Rayk Behrendt, Tina Schumann, Alexander Gerbaulet, Laura A. Nguyen, Nadja Schubert, Dimitra Alexopoulou, Ursula Berka, Stefan Lienenklaus, Katrin Peschke, Kathrin Gibbert, Sabine Wittmann, Dirk Lindemann, Siegfried Weiss, Andreas Dahl, Ronald Naumann, Ulf Dittmer, Baek Kim, Werner Mueller, Thomas Gramberg, Axel Roers

**Affiliations:** 1; 2; 3; 4; 5; 6; 7; 8; 9; 10; 11

## Abstract

Aicardi-Goutières syndrome (AGS), a hereditary autoimmune disease, clinically and biochemically overlaps with systemic lupus erythematosus (SLE) and, like SLE, is characterized by spontaneous type I interferon (IFN) production. The finding that defects of intracellular nucleases cause AGS led to the concept that intracellular accumulation of nucleic acids triggers inappropriate production of type I IFN and autoimmunity. AGS can also be caused by defects of SAMHD1, a 3^′^ exonuclease and deoxy-nucleotide (dNTP) triphosphohydrolase. Human SAMHD1 is an HIV-1 restriction factor that hydrolyzes dNTPs and decreases their concentration below the levels required for retroviral reverse transcription. We show in gene-targeted mice that also mouse SAMHD1 reduces cellular dNTP concentrations and restricts retroviral replication in lymphocytes, macrophages, and dendritic cells. Importantly, the absence of SAMHD1 triggered IFN-β-dependent transcriptional upregulation of type I IFN-inducible genes in various cell types indicative of spontaneous IFN production. SAMHD1-deficient mice may be instrumental for elucidating the mechanisms that trigger pathogenic type I IFN responses in AGS and SLE.

## INTRODUCTION

Systemic lupus erythematosus (SLE) is characterized by a chronic type I interferon (IFN) response, which is considered to play a central role in the breakdown of self-tolerance (Banchereau and Pascual, 2006; Pascual et al., 2006; Rönnblom et al., 2011). Defective clearance of chromatin-containing cellular debris resulting in uncontrolled activation of sensors of the innate immune system by endogenous nucleic acids has been extensively discussed as a mechanism that triggers a pathogenic IFN response. Aicardi-Goutières syndrome (AGS) is characterized by early-onset leukencephalopathy resembling congenital viral infection with intracerebral calcifications and cerebrospinal fluid lymphocytosis (Crow and Rehwinkel, 2009). Importantly, AGS clinically overlaps with SLE and, like SLE, features uncontrolled production of type I IFN, although to date, no viral infection has been causally linked to AGS (Rice et al., 2007b). AGS can be caused by mutations in one of four intracellular enzymes involved in nucleic acid metabolism, including 3^′^ repair exonuclease (TREX)1 (Crow et al., 2006a), a 3^′^ exonuclease with substrate preference for single-stranded DNA (ssDNA); RNase H2 (Crow et al., 2006b), which cleaves RNA:DNA hybrids; adenosine deaminase acting on RNA 1 (ADAR1) (Rice et al., 2012), an RNA editing enzyme; and the deoxynucleotide triphosphohydrolase SAM domain and HD domain 1 (SAMHD1) (Rice et al., 2009). The finding that a rare monogenic form of lupus can also be caused by mutations in TREX1 (Lee-Kirsch et al., 2007a; Rice et al., 2007a) or SAMHD1 (Ravenscroft et al., 2011), as well as the identification of SLE patients carrying mutations in TREX1 (Lee-Kirsch et al., 2007b) and a report of the coexistence of AGS and SLE in a patient with mutated SAMHD1 (Ramantani et al., 2011), further underpinned the tight pathogenetic relationship between AGS and SLE.

*Trex1*^−^*^/^*^−^ mice spontaneously develop autoimmune multiorgan inflammation, which can be rescued by additional inactivation of the type I IFN system, demonstrating the critical pathogenetic role of the IFN response (Morita et al., 2004; Stetson et al., 2008; Gall et al., 2012). These findings led to a new concept of autoimmunity caused by defects of nucleic acid metabolism resulting in intracellular accumulation of endogenous nucleic acids, which in turn trigger a type I IFN response (Stetson et al., 2008). Short ssDNA species originating during DNA replication (Yang et al., 2007) or replication intermediates of endogenous retroelements (Stetson et al., 2008) were proposed to be responsible for activation of the IFN system in *Trex1*^−^*^/^*^−^ mice. Loss of ADAR1 in mice is embryonically lethal and *Adar1*^−^*^/^*^−^ mouse embryos, like *Trex1*^−^*/*^−^ mice, display spontaneous production of type I IFN (Hartner et al., 2009). Mice deficient for RNase H2 accumulate ribonucleotides in genomic DNA, resulting in a spontaneous DNA damage response (Reijns et al., 2012; Hiller et al., 2012). The relevance of this observation for the induction of autoimmunity in AGS and SLE is presently unclear.

SAMHD1 contains a sterile alpha motif (SAM) and a motif of cation-coordinating His and Asp residues (HD domain) that are encountered in proteins with functions in nucleic acid metabolism. Recently, SAMHD1 was demonstrated to restrict HIV-1 replication by hydrolyzing dNTPs to deoxynucleosides and inorganic triphosphate in a deoxyguanosine triphosphate (dGTP)-dependent fashion. This results in reduction of cellular dNTP concentrations below the levels required for retroviral reverse transcription (Hrecka et al., 2011; Laguette et al., 2011; Lahouassa et al., 2012; Goldstone et al., 2011; Powell et al., 2011; Kim et al., 2012). SAMHD1-mediated dNTP reduction is operative in noncycling myeloid cells as well as in resting T cells, and is at least partly responsible for the relative resistance of dendritic cells and macrophages to HIV-1 infection (Baldauf et al., 2012; Descours et al., 2012; Berger et al., 2011). The Vpx accessory protein of many SIV strains and HIV-2 counteracts this restriction by targeting SAMHD1 for proteasomal degradation (Hrecka et al., 2011; Laguette et al., 2011). The antiretroviral activity is regulated by phosphorylation of SAMHD1 at Thr592 by Cyclin A2/CDK1 (Cribier et al., 2013; White et al., 2013b). In addition to its deoxynucleotide hydrolase activity, SAMHD1 was recently shown to directly interact with nucleic acids and to exhibit 3^′^ exonuclease activity against RNA and DNA in vitro (Beloglazova et al., 2013; Tüngler et al., 2013).

Herein, we show that mouse SAMHD1, like the human enzyme, decreases the dNTP pool in murine cells and restricts retroviral infection. Importantly, we found that SAMHD1-deficient mice display spontaneous type I IFN production and thus model an important feature of AGS and SLE pathogenesis.

## RESULTS

### SAMHD1-Deficient Mice

Targeted embryonic stem cells (ESCs) carrying a gene trap cassette inserted into *intron1* of the *SAMHD1* gene were used to generate *SAMHD1 knockout first* (*KOF*) mice (Skarnes et al., 2011; Figure S1). The gene trap cassette was spliced to *exon1* as expected (Figure S1). A cryptic splice donor site 115 bp downstream of the gene trap splice acceptor was used to splice to *exon2* resulting in a frameshift and premature stops (Figure S1). Low levels of the mutant sterile transcript could be detected, whereas wild-type (WT) messenger RNA (mRNA) was absent in *SAMHD1^KOF/KOF^* cells (Figure S1). A second SAMHD1-deficient mouse strain, *SAMHD1*^Δ/Δ^, was generated by in vivo deletion of the gene trap cassette and *loxP*-flanked *exon2* (by Flpe-and Cre-mediated recombination, respectively; Figure S1). In *SAMHD1*^Δ/Δ^ mice, a frameshift precludes gene expression. Absence of SAMHD1 protein in *SAMHD1^KOF/KOF^* and *SAMHD1*^Δ^*^/^*^Δ^ mice was demonstrated by western blot (Figure S1). *SAMHD1^KOF/KOF^* and *SAMHD1*^Δ^*^/^*^Δ^ mice were obtained in Mendelian ratios (not shown). Both strains were fertile and did not feature macroscopic abnormalities.

### Murine SAMHD1 Decreases Cellular dNTP Concentrations and Restricts Retroviral Replication

**Figure 1:**
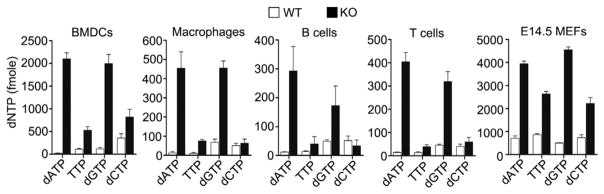
Murine SAMHD1 Functions to Reduce Cellular dNTP Concentrations An assay based on incorporation of a spe-cific single nucleotide into a radiolabeled template by RT was used to quantify the relative amounts of each nucleotide in *SAMHD1*^Δ^*^/^*^Δ^ (KO) or control cells (WT). The indicated cell types were flow-cytometrically sorted (except for E14.5 MEFs), followed by extraction of dNTPs, which were subsequently used as substrates for the individual in vitro RT reactions. In a second independent experiment, two dNTP extracts of pools from peritoneal macrophages and one pool from bone-marrow-derived macrophages from *SAMHD1*^Δ^*^/^*^Δ^ or control cells yielded similar results. Displayed are the means ± SD of two independent measurements, each performed in triplicate.

Human SAMHD1 blocks HIV-1 replication in myeloid cells by reducing the concentration of cellular dNTPs below the threshold level required for reverse transcription (Hrecka et al., 2011; Laguette et al., 2011). To test whether murine SAMHD1 also deprives cells of dNTPs, we compared dNTP levels in primary macrophages, bone-marrow-derived dendritic cells (BMDCs), splenic B and T cells, and embryonic day 14.5 (E14.5) mouse embryonic fibroblasts (MEFs) from *SAMHD1*^Δ^*^/^*^Δ^ mice and littermate control mice. In all cell types derived from SAMHD1-deficient mice, the concentration of dNTPs was substantially higher than in WT cells (Figure 1). Thus, murine SAMHD1 functions to reduce the intracellular concentration of dNTPs.

**Figure 2:**
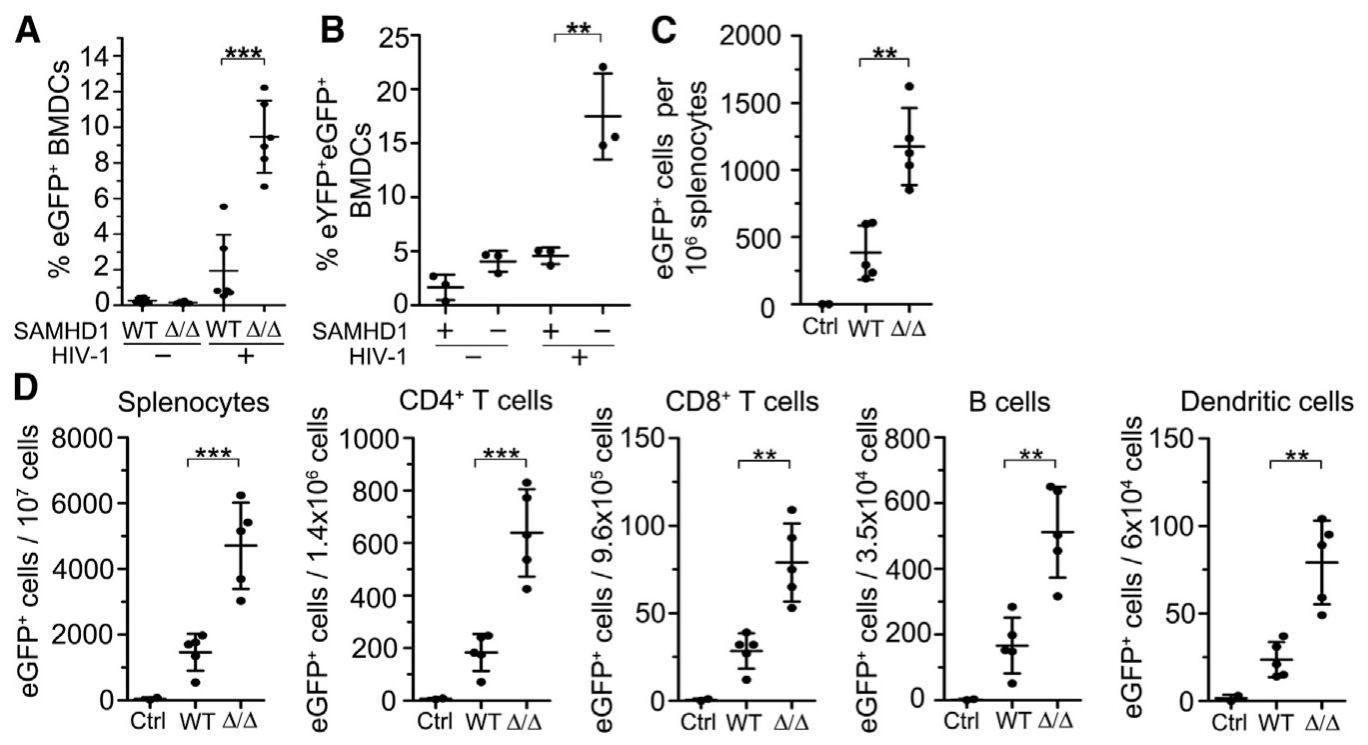
Murine SAMHD1 Restricts Retro-viral Replication (A) BMDCs were generated from six mutant and six control mice and infected with EGFP-containing HIV-1-VSV-G (VSVG/NL43-CMVGFP). Cellular EGFP fluorescence, indicating that the virus had successfully reverse transcribed and integrated its genome, was detected by FACS. The graph represents a summary of two independent experiments with similar results. A third experiment with cells from three additional mutant and three additional control mice yielded similar results (Figure S2). Means ± SD are displayed. (B) BMDCs from three SAMHD1-deficient mice were transduced with a lentiviral vector carrying either an EYFP and a murine SAMHD1 expression cassette or only the EYFP cassette. After 48 hr, the transduced cells were challenged with the EGFP-expressing HIV-1 pseudotyped with VSV-G (see A). EYFP^+^ cells were analyzed by FACS for EGFP expression 48 hr after challenge. Means ± SD are displayed. (C) Five *SAMHD1*^Δ^*^/^*^Δ^ and five *SAMHD1^WT/WT^* mice were injected intravenously with 1.8 × 10^7^ particles of an EGFP-expressing HIV-1 pseudotyped with VSV-G (VSVG/HR.CMVGFP) and two control mice (Ctrl, one *SAMHD1*^Δ^*^/^*^Δ^ and one *SAMHD1^WT/WT^*) were injected with PBS. After 3 days, the mice were sacrificed and the absolute numbers of live EGFP^+^ cells per 10^6^ splenocytes were determined by flow cytometry. Means ± SD are displayed. Differential analysis of individual cell populations from this experiment for HIV reporter infection is shown in Figure S2. (D) Infection of five additional *SAMHD1*^Δ^*^/^*^Δ^ and five additional WT mice with 5 × 10^6^ particles of HIV reporter virus as in (C). Absolute numbers of live EGFP^+^ cells in different splenic cell populations are shown. Means ± SD are displayed. See also Figure S2.

Next, we sought to address whether SAMHD1-mediated dNTP reduction results in restriction of retroviral replication in murine cells and in mice in vivo. Therefore, we infected BMDCs from SAMHD1-deficient and control mice with an HIV-1 reporter virus in vitro. A single-cycle HIV-1 reporter virus based on the HIV clone NL4-3 (VSV-G/NL43-CMVGFP) was generated by transfection of proviral DNA into 293T cells. Viral particles encoding a cytomegalovirus (CMV) promotor-driven EGFP reporter gene were pseudotyped with the glycoprotein of vesicular stomatitis virus (VSV-G). Upon infection of the target cell, the viral RNA genome is reverse transcribed and transported into the nucleus where EGFP is expressed from integrated provirus. We found that upon infection of murine BMDCs, the frequency of EGFP-expressing cells was ~5-fold higher in *SAMHD1*^Δ^*^/^*^Δ^ cells compared with control cells, as shown in Figure 2A, which represents a summary of two independent experiments. A third experiment based on the same virus (Figure S2) and an additional experiment using a different NL4-3-based reporter virus (VSV-G/HR.CMVGFP, not shown) yielded similar results. To verify that the enhanced infection of *SAMHD1*^Δ^*^/^*^Δ^ BMDCs was due to the absence of SAMHD1 activity, we complemented *SAMHD1*^Δ^*^/^*^Δ^ cells by transduction with lentiviral particles encoding murine SAMHD1 or empty vector prior to infection with NL43-CMVGFP. In contrast to control vector-transduced cells, we found that reconstituted SAMHD1 restored HIV-1 restriction in otherwise permissive SAMHD1-defi-cient BMDCs (Figure 2B). Infection of *SAMHD1*^Δ^*^/^*^Δ^ and control BMDCs with a third HIV reporter virus based on a different isolate (HXB2) showed moderate SAMHD1-mediated restriction in some experiments, but not in others (not shown).

In order to address retroviral restriction by SAMHD1 also in vivo, we infected SAMHD1-deficient and control mice with an NL4-3-based VSV-G/HR.CMVGFP reporter virus by intravenous injection and determined the frequencies of EGFP-expressing cells 3 days later. As shown in Figure 2C, ~4-fold higher numbers of EGFP-expressing cells were detected in SAMHD1-deficient versus control splenocytes. In a second, independent experiment, the number of EGFP^+^ splenocytes was 6-fold increased in mutant versus control mice (Figure 2D, left panel). Differential analysis of the frequencies of EGFP^+^ cells in individual splenic cell populations from these two experiments revealed that SAMHD1-mediated retroviral restriction was operative in CD4^+^ and CD8^+^ T cells, B cells, dendritic cells, and macrophages (Figures 2D and S2B). In contrast, the course of in vivo infection with Friend virus (FV), a murine leukemia virus (MLV), was not different in *SAMHD1*^Δ^*^/^*^Δ^ versus control mice (Figure S2). We speculate that the replication of FV might not be susceptible to restriction by SAMHD1 since the FV life cycle requires cell division, whereas SAMHD1-mediated antiviral activity seems to be limited to noncycling cells in which dNTP pools are generally lower compared with cells in cycle (Baldauf et al., 2012; Descours et al., 2012; Rampazzo et al., 2010). However, restriction of different MLVs by SAMHD1 was recently reported in noncycling phorbol myristate acetate-differentiated human U937 cells in vitro (White et al., 2013a, 2013b).

Collectively, our results show that mouse SAMHD1 reduces cellular dNTP concentrations and inhibits retroviral replication. The susceptibility to restriction by SAMHD1 differs among different classes of retroviruses, and even different isolates of the same virus may differ in their susceptibility to low dNTP levels.

### Spontaneous Type I IFN Release in SAMHD1-Deficient Cells

**Figure 3:**
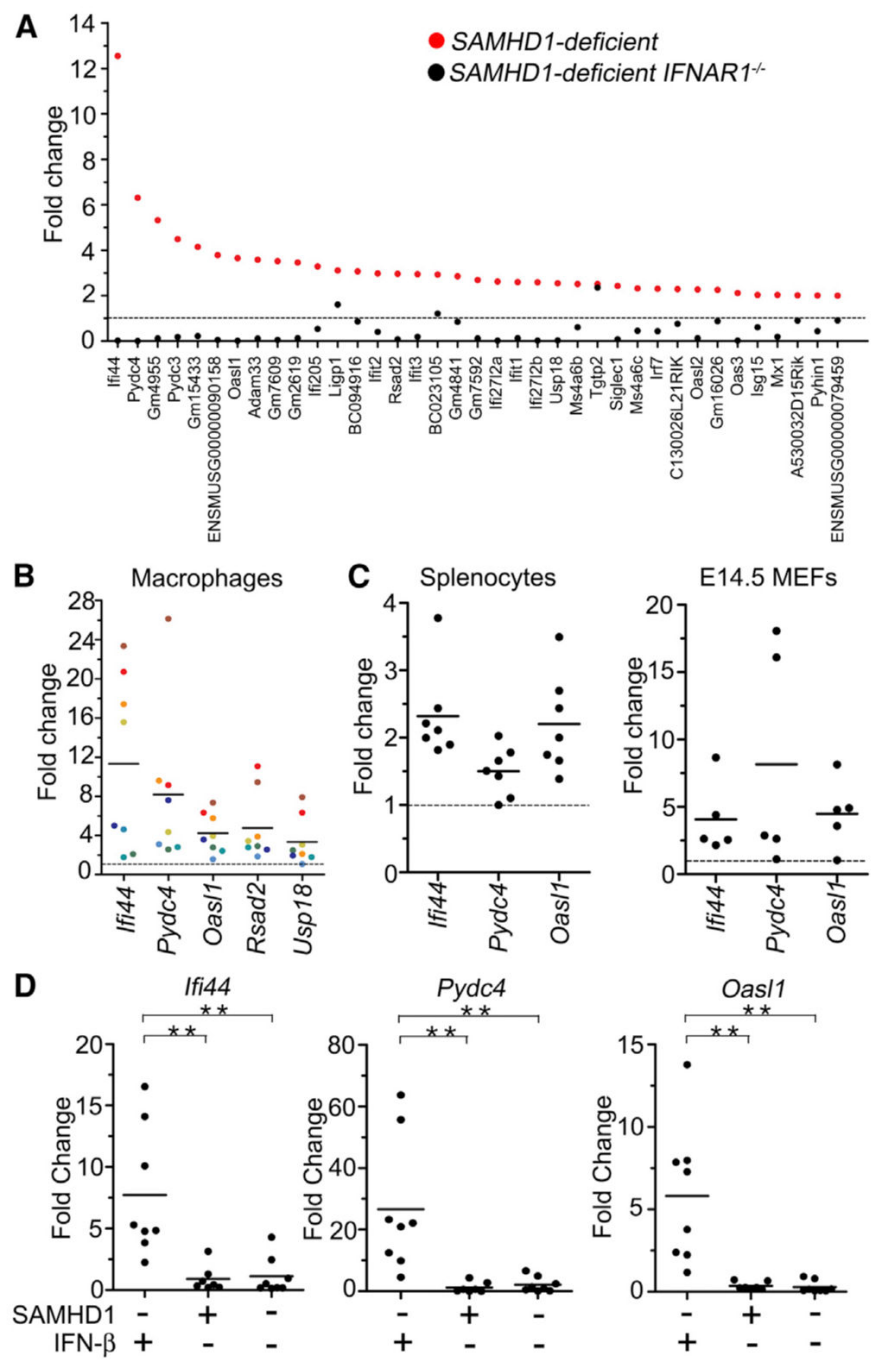
Spontaneous Induction of Type I IFN-Inducible Genes in SAMHD1-Deficient Cells (A) mRNA of FACS-sorted peritoneal macrophages from ten SAMHD1-defi-cient, ten SAMHD1-deficient *IFNAR1*^−^*/*^−^, and ten control mice was analyzed by Illumina-based transcriptome sequencing. In total, 37 genes were upregulated at least 2-fold (p < 0.05) in SAMHD1-deficient mice compared with controls. All but two of these genes were known to be inducible by type I IFN (displayed as red dots). In the comparison of SAMHD1-deficient *IFNAR1*^− /−^ versus control cells, reduced transcript levels, rather than upregulation, were observed for most of these genes (black dots). (B) Transcript levels of five IFN-inducible genes found to be upregulated in the transcriptome analysis (A) were quantified in peritoneal macrophages from eight additional *SAMHD1*-deficient and 18 additional control mice by qRT-PCR. Fold upregulation for individual mutant samples compared with the mean of all control samples is displayed with colors identifying individual mice. (C) Transcript levels of three IFN-inducible genes were assessed in SAMHD1-deficient (n = 7) and control (n = 4) splenocytes, and in mutant (n = 5) and control (n = 4) E14.5 MEFs by qRT-PCR. Results are displayed as fold change for each mutant compared with the means of all controls. All genes were significantly upregulated (p < 0.04) in all mutant samples analyzed. (D) Transcript levels of three IFN-inducible genes in peritoneal macrophages from *SAMHD1*^Δ^*^/^*^Δ^ IFN-β-deficient (n = 8), *SAMHD1*^Δ^*^/^*^Δ^ IFN-βcompetent (n = 8), and *SAMHD1^WT/WT^* IFN-β-deficient (n = 8) mice as determined by qRT-PCR. Results are displayed as fold change for each individual sample compared with the mean of all WT control samples (n = 8). See also Figure S3.

Defects of two other AGS-associated enzymes, TREX1 and ADAR1, result in spontaneous production of type I IFN in mice. To investigate whether loss of SAMHD1 also triggers activation of an antiviral response in the mouse, we compared global gene-expression profiles of cells from SAMHD1-deficient and control mice. Because SAMHD1-mediated restriction was prominent in myeloid cells, we chose to flow-cytometrically sort macrophages from peritoneal lavage fluid (Figure S3). We performed three independent experiments including a total of 25 SAMHD1-deficient (*SAMHD1*^Δ^*^/^*^Δ^ or *SAMHD1^KOF/KOF^*) and 19 control mice. In the first experiment, RNA from ten mutant and ten control mice was subjected to Illumina-based transcriptome sequencing (RNA-Seq). We found that 141 genes were significantly (padj < 0.05) upregulated in SAMHD1-deficient compared with control macrophages, and 123 (85%) of these were reported to be inducible by type I IFN in the literature (Figure 3A; Table S1). A second experiment (Figure S3) and a third experiment (not shown) including a total of 15 mutant and nine control samples yielded very similar results. Macrophages from an additional cohort of ten SAMHD1-deficient mice that were also deficient for the type I IFN receptor (*SAMHD1^KOF/KOF^IFNAR1*^−^*^/^*^−^ mice) did not display the upregulation of IFN-inducible genes observed in SAMHD1-deficient mice with intact type I IFN receptor. Instead, *SAMHD1^KOF/KOF^IFNAR1*^−^*^/^*^−^ macrophages featured a downregulation of IFN-inducible genes compared with WT control cells. Hasan et al. (2013) showed that the induction of antiviral genes in *Trex1*^−^*^/^*^−^ mice can occur in an IFN-independent fashion in particular cell types. Our findings demonstrate that the spontaneous transcriptional signature of antiviral genes in SAMHD1-deficient macrophages is induced by type I IFN. Hasan et al. (2013) did not observe a transcriptional signature of IFN-inducible genes in fibroblasts from an AGS patient carrying the mutation *SAMHD1^R290H/Q548X^*, implying that spontaneous activation of IFN production may represent a cell-type-specific phenomenon as suggested for TREX1 deficiency in mice (Gall et al., 2012). In addition to Illumina-based transcriptome profiling, we confirmed upregulation of five genes (*Ifi44*, *Pydc4*, *Oasl1*, *Rsad2*, and *Usp18*) by quantitative RT-PCR (qRT-PCR) of total RNA from fluorescence-activated cell sorting (FACS)-purified peritoneal macrophages of eight additional SAMHD1-deficient and 18 additional control mice. In all mutant samples, levels of the five transcripts were increased compared with the mean of controls (Figure 3B). Spontaneous upregulation of IFN-inducible genes was also detected in SAMHD1-deficient splenocytes and E14.5 MEFs compared with control cells (Figure 3C). Crossing *SAMHD1*^Δ^*^/^*^Δ^ mice onto an IFN-β-deficient background (see Experimental Procedures) prevented spontaneous transcriptional activation of type I IFN-inducible genes, as shown by qRT-PCR on three genes (*Ifi44*, *Pydc4*, and *Oasl1*; Figure 3D). This demonstrates that IFN-β is indispensible for the spontaneous activation of IFN-inducible genes in SAMHD1-deficient mice and corroborates our findings in SAMHD1-deficient *IFNAR1*^−^*^/^*^−^ macrophages.

## DISCUSSION

Our findings thus indicate that an important function of SAMHD1 is to suppress spontaneous activation of the type I IFN system in the steady state. AGS can be caused by defects in one of four intracellular enzymes with functions in nucleic acid metabolism: TREX1, RNase H2, ADAR1, and SAMHD1. AGS clinically overlaps with SLE, and both diseases are associated with a spontaneous antiviral type I IFN response in the absence of detectable exogenous virus infection. Intriguingly, TREX1 was reported to have an impact on HIV infection by degrading nonproductive by-products of reverse transcription (Yan et al., 2010). These DNA fragments, although useless for HIV replication, can be sensed by the infected cell and induce antiviral IFN responses. Their degradation by TREX1 serves to hide HIV from the innate immune system, preventing the IFN response. ADAR1 was shown to promote retroviral replication in some studies (Phuphuakrat et al., 2008; Doria et al., 2009), but was described to have antiretroviral activity in a recent report (Biswas et al., 2012). SAMHD1 is an important HIV-1 restriction factor in noncycling human cells, which inhibits reverse transcription by decreasing dNTP concentrations (Hrecka et al., 2011; Laguette et al., 2011; Lahouassa et al., 2012; Goldstone et al., 2011; Powell et al., 2011; Kim et al., 2012). Thus, defects of one of several cellular enzymes that affect retroviral infection can cause human autoimmune disease associated with uncontrolled type I IFN responses. A causative link to an exogenous viral infection in these conditions has not been established. The above findings fuel the attractive hypothesis that AGS-associated enzymes are important restriction factors for the activity of endogenous retro-viruses and/or retrotransposons in the steady state. Loss of these restriction factors may result in intracellular accumulation of nucleic acids derived from these endogenous retroelements and lead to their detection by intracellular nucleic acid sensors. The ensuing cell-intrinsic innate antiviral response entails chronic production of type I IFN, which in turn leads to autoimmunity. Stetson et al. (2008) detected increased frequencies of retroelement-encoded DNA sequences in the cytosol of *Trex1*^−^*^/^*^−^ cells, providing evidence for enhanced activity of retroelements in the absence of TREX1. Intriguingly, a combination of HIV-1 reverse transcriptase (RT) inhibitors, which are also used to treat HIV infection, significantly ameliorated the pathology of *Trex1*^−^*^/^*^−^ mice (Beck-Engeser et al., 2011). Rather than replication intermediates of endogenous retroelements, Yang et al. (2007) proposed nucleic acids arising during replication of the cellular genome as stimulators of the pathogenic IFN response in *Trex1*^−^*^/^*^−^ mice.

**Figure 4:**
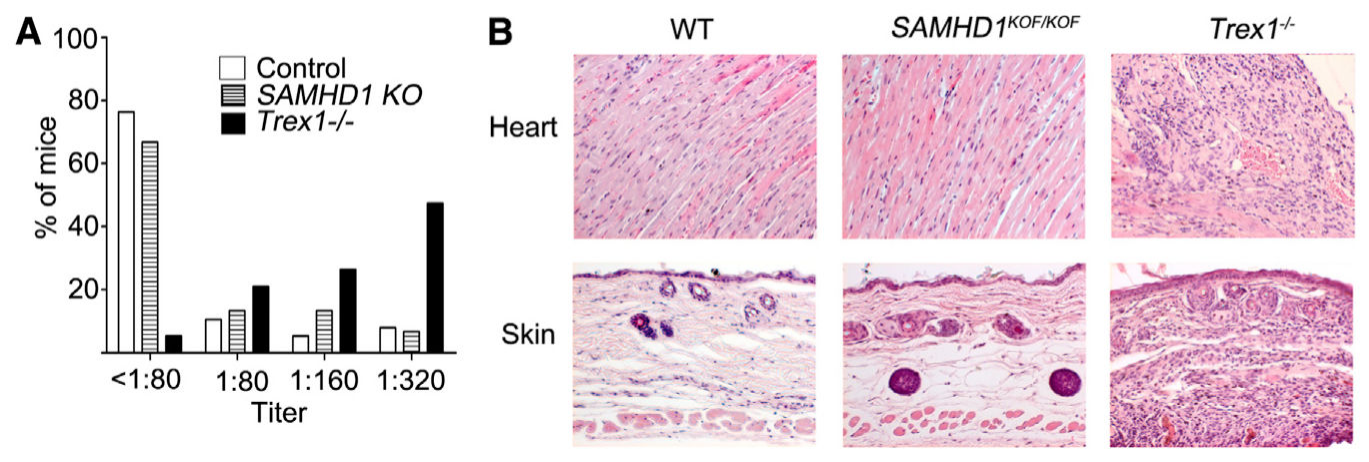
Spontaneous IFN Release Does Not Result in Autoimmune Disease in SAMHD1-Deficient Mice (A) ANAs were detected by immunofluorescence analysis on murine fibroblasts. The significance of differences in ANA prevalence between groups was tested by chi-square test (Pearson) with Bonferroni correction. Autoantibodies occurred more frequently in *Trex1*^−^*/*^−^ mice (n = 19, mean age 10 weeks) compared with controls (Ctrl, p < 0.0001, n = 38, mean age 15 weeks) or SAMHD1-deficient mice (SAMHD1 KO, p < 0.005, n = 15 [this group comprised *SAMHD1^KOF/KOF^* and *SAMHD1*^Δ^*^/^*^Δ^ mice, mean age 20 weeks]). No significant increase in ANA frequencies could be detected in the SAMHD1-deficient versus the control group. (B) Absence of inflammatory changes in heart and skin from SAMHD1-deficient mice. H&E-stained sections of heart and skin from one WT, one *SAMHD1^KOF/KOF^* animal, and one diseased *Trex1*^−^*/*^−^ animal from our facility, representative of 13 *SAMHD1^KOF/KOF^* mice analyzed (2 mice 16 weeks old, 11 older than 53 weeks) and five *SAMHD1*^Δ^*^/^*^Δ^ mice (3 mice 9–15 weeks old, 2 older than 43 weeks). See also Figure S4.

Knockout mouse models for TREX1 and ADAR1 are both characterized by lethal phenotypes, both of which are associated with spontaneous production of type I IFN. The systemic autoimmune pathology of *Trex1*^−^*^/^*^−^ mice is reverted by additional inactivation of the genes encoding interferon regulatory factor 3 (IRF3) or the type I IFN receptor, demonstrating the pivotal role of the IFN response in the pathogenetic chain. Our findings add SAMHD1 to the list of AGS-associated enzymes, defects of which trigger spontaneous type I IFN production in the mouse. In contrast to *Trex1*^−^*/*^−^ and *Adar1*^−^*/*^−^ mice (Morita et al., 2004; Hartner et al., 2009), SAMHD1-deficient mice did not develop detectable pathology or autoimmunity. The animals did not develop macroscopic abnormalities up to the age of 70 weeks. Histological analysis of tissues affected in AGS patients and *Trex1*^−^*/*^−^ mice, including heart, skin (Figure 4), lung, liver, kidney, large arteries, skeletal muscle, skin, and brain (not shown), did not reveal inflammatory changes in *SAMHD1^KOF/KOF^* (n = 13 [2 mice 16 weeks old, 11 older than 53 weeks]) or *SAMHD1*^Δ^*^/^*^Δ^ mice (n = 5 [3 mice 9–15 weeks old, 2 older than 43 weeks]). Both AGS patients and *Trex1*^−^*^/^*^−^ mice show a high incidence of antinuclear antibodies (ANAs) (Gall et al., 2012; Ramantani et al., 2010). The incidence of ANAs in SAMHD1-deficient mice, however, was not different from that in control C57BL/6 mice under the conditions of our animal facility, whereas ANAs in titers above 1:320 (Figure 4), as well as lethal pathology (Figure S4), developed frequently in our cohort of *Trex1*^−^*^/^*^−^ animals as expected. We speculate that levels of spontaneously released type I IFN range below a threshold required for breakdown of self-tolerance. Nevertheless, it seems likely that the activation of the IFN response in SAMHD1-deficient mice reflects an important pathogenic event of SAMHD1-associated AGS.

Our experiments did not address the question of cellular sources of type I IFN in SAMHD1-deficient mice. It will be important to compare cell types that produce IFN in the various knockout mouse models of AGS-associated enzymes. Moreover, the innate sensors that trigger the spontaneous IFN response of SAMHD1- and ADAR1-deficient cells remain unknown; however, the pathology of *Trex1*^−^*^/^*^−^ mice was demonstrated to depend on the STING/TBK1/IRF3 pathway (Gall et al., 2012).

In conclusion, we have identified mouse SAMHD1 as a retro-viral restriction factor that reduces cellular dNTP pools. Moreover, SAMHD1-deficient mice display a spontaneous activation of the type I IFN system and thus reproduce an important feature of AGS pathogenesis. SAMHD1 mutant mice may therefore be instrumental in elucidating the mechanisms that trigger pathogenic type I IFN responses in AGS and SLE.

## EXPERIMENTAL PROCEDURES

### Mice

Generation of SAMHD1-deficient mice was based on a targeted C57BL/6 JM8A3.N1 ESC clone (EPD0424_3_A06; http://www.knockoutmouse.org). Mice deficient for IFN-β (Δβ*-luc*; Lienenklaus et al., 2009) were on the C57BL/6 background. TREX1-deficient and *IFNAR1*^−^*^/^*^−^ mice (C57BL/6 background; Morita et al., 2004) were kindly provided by Thomas Lindahl (London Research Institute, London, UK) and Ulrich Kalinke (TWINCORE, Hannover, Germany), respectively. The mice were kept under specific pathogen-free conditions at the Experimental Center of the University of Technology Dresden. All animal experiments were done according to institutional guidelines on animal welfare and were approved by the Landesdirektion Dresden.

### Immunofluorescence for Detection of ANA

3T3 cells were fixed on glass slides and incubated with diluted sera for 30 min. Following two washes with PBS with 0.05% (v/v) Tween 20, slides were incubated for 30 min in a 1:100 dilution of a sheep-anti-mouse-IgG-FITC antibody-conjugate (Seramun Diagnostica) in PBS. After two washes, the slides were covered with embedding media (Euroimmun AG) and analyzed.

### dNTP Assay

A nucleotide incorporation assay was conducted as described previously (Diamond et al., 2004).

### In Vitro and In Vivo HIV Reporter Virus Infection

For in vitro HIV-1 infection assays, 3.0 × 10^5^ BMDCs from *SAMHD1*^Δ^*^/^*^Δ^ and WT mice were infected with HIV-1-GFP at a multiplicity of infection (moi) = 1. The number of infected cells was determined 3 days later by flow cytometry. For SAMHD1-complementation assays, 1.0 × 10^5^ BMDCs from *SAMHD1*^Δ^*^/^*^Δ^ mice were transduced with 50 ng p24 of SAMHD1-internal ribosome entry site (IRES)-yellow fluorescent protein (YFP) viral vectors or control particles. After 3 days, cells were infected with HIV-1-GFP at a moi = 1. The number of infected cells was determined 3 days later by flow cytometry.

Mice were injected intravenously with 0.5 ml PBS or PBS containing 1.8 × 10^7^ (first experiment) or 5 × 10^6^ (second experiment) particles of pseudotyped HIV-1 reporter virus (VSV-G/HR.CMVGFP; see above). After 3 days, the frequencies of propidium iodide-negative EGFP^+^ cells in total splenocytes, CD4^+^ and CD8^+^ T cells, B220^+^ B cells, CD11b^+^ CD11c^+^ dendritic cells, and CD11b^+^ F4/80^+^ macrophages were analyzed by flow cytometry (antibodies from eBioscience and BioLegend).

### Sorting of Peritoneal Macrophages for Illumina-Based Transcriptome Analysis

CD11b^+^ F4/80^+^ peritoneal macrophages (antibodies from eBioscience and BioLegend, respectively) were flow-cytometrically sorted from peritoneal lavage of *SAMHD1*^Δ^*^/^*^Δ^ and WT mice, and lysed immediately in *μ*MACS mRNA Isolation Kit lysis buffer (Miltenyi Biotec).

### Statistical Analysis

If not stated otherwise, data are shown as means ± SD. Statistical analysis was performed using a Student’s t test (unpaired t test, two-tailed, 95% confidence intervals). Significance levels in each figure are indicated by asterisks (*p ≤ 0.05, **p < 0.01, ***p < 0.001).

For further details regarding the materials and methods used in this study, see the Extended Experimental Procedures.

## Supplementary Material

Extended Experimental Procedures and Supplemental Figures

Table S1

## References

[R1] Anders S, Huber W (2010). Differential expression analysis for sequence count data. Genome Biol.

[R2] Baldauf HM, Pan X, Erikson E, Schmidt S, Daddacha W, Burggraf M, Schenkova K, Ambiel I, Wabnitz G, Gramberg T (2012). SAMHD1 restricts HIV-1 infection in resting CD4(+) T cells. Nat Med.

[R3] Banchereau J, Pascual V (2006). Type I interferon in systemic lupus erythematosus and other autoimmune diseases. Immunity.

[R4] Beck-Engeser GB, Eilat D, Wabl M (2011). An autoimmune disease prevented by anti-retroviral drugs. Retrovirology.

[R5] Beloglazova N, Flick R, Tchigvintsev A, Brown G, Popovic A, Nocek B, Yakunin AF (2013). Nuclease activity of the human SAMHD1 protein implicated in the Aicardi-Goutieres syndrome and HIV-1 restriction. J Biol Chem.

[R6] Berger A, Sommer AF, Zwarg J, Hamdorf M, Welzel K, Esly N, Panitz S, Reuter A, Ramos I, Jatiani A (2011). SAMHD1-deficient CD14+ cells from individuals with Aicardi-Goutières syndrome are highly susceptible to HIV-1 infection. PLoS Pathog.

[R7] Biswas N, Wang T, Ding M, Tumne A, Chen Y, Wang Q, Gupta P (2012). ADAR1 is a novel multi targeted anti-HIV-1 cellular protein. Virology.

[R8] Cribier A, Descours B, Valadao AL, Laguette N, Benkirane M (2013). Phosphorylation of SAMHD1 by cyclin A2/CDK1 regulates its restriction activity toward HIV-1. Cell Rep.

[R9] Crow YJ, Rehwinkel J (2009). Aicardi-Goutieres syndrome and related phenotypes: linking nucleic acid metabolism with autoimmunity. Hum Mol Genet.

[R10] Crow YJ, Hayward BE, Parmar R, Robins P, Leitch A, Ali M, Black DN, van Bokhoven H, Brunner HG, Hamel BC (2006a). Mutations in the gene encoding the 3^′^-5^′^ DNA exonuclease TREX1 cause Aicardi-Goutières syndrome at the AGS1 locus. Nat Genet.

[R11] Crow YJ, Leitch A, Hayward BE, Garner A, Parmar R, Griffith E, Ali M, Semple C, Aicardi J, Babul-Hirji R (2006b). Mutations in genes encoding ribonuclease H2 subunits cause Aicardi-Goutières syndrome and mimic congenital viral brain infection. Nat Genet.

[R12] Descours B, Cribier A, Chable-Bessia C, Ayinde D, Rice G, Crow Y, Yatim A, Schwartz O, Laguette N, Benkirane M (2012). SAMHD1 restricts HIV-1 reverse transcription in quiescent CD4(+) T-cells. Retrovirology.

[R13] Diamond TL, Roshal M, Jamburuthugoda VK, Reynolds HM, Merriam AR, Lee KY, Balakrishnan M, Bambara RA, Planelles V, Dewhurst S, Kim B (2004). Macrophage tropism of HIV-1 depends on efficient cellular dNTP utilization by reverse transcriptase. J Biol Chem.

[R14] Dittmer U, Brooks DM, Hasenkrug KJ (1998). Characterization of a live-attenuated retroviral vaccine demonstrates protection via immune mechanisms. J Virol.

[R15] Doria M, Neri F, Gallo A, Farace MG, Michienzi A (2009). Editing of HIV-1 RNA by the double-stranded RNA deaminase ADAR1 stimulates viral infection. Nucleic Acids Res.

[R16] Gall A, Treuting P, Elkon KB, Loo YM, Gale M, Barber GN, Stetson DB (2012). Autoimmunity initiates in nonhematopoietic cells and progresses via lymphocytes in an interferon-dependent autoimmune disease. Immunity.

[R17] Goldstone DC, Ennis-Adeniran V, Hedden JJ, Groom HC, Rice GI, Christodoulou E, Walker PA, Kelly G, Haire LF, Yap MW (2011). HIV-1 restriction factor SAMHD1 is a deoxynucleoside triphosphate triphosphohydrolase. Nature.

[R18] Habegger L, Sboner A, Gianoulis TA, Rozowsky J, Agarwal A, Snyder M, Gerstein M (2011). RSEQtools: a modular framework to analyze RNA-Seq data using compact, anonymized data summaries. Bioinformatics.

[R19] Hartner JC, Walkley CR, Lu J, Orkin SH (2009). ADAR1 is essential for the maintenance of hematopoiesis and suppression of interferon signaling. Nat Immunol.

[R20] Hasan M, Koch J, Rakheja D, Pattnaik AK, Brugarolas J, Dozmorov I, Levine B, Wakeland EK, Lee-Kirsch MA, Yan N (2013). Trex1 regulates lysosomal biogenesis and interferon-independent activation of antiviral genes. Nat Immunol.

[R21] Hasenkrug KJ, Brooks DM, Robertson MN, Srinivas RV, Chesebro B (1998). Immunoprotective determinants in friend murine leukemia virus envelope protein. Virology.

[R22] Hiller B, Achleitner M, Glage S, Naumann R, Behrendt R, Roers A (2012). Mammalian RNase H2 removes ribonucleotides from DNA to maintain genome integrity. J Exp Med.

[R23] Hofmann H, Logue EC, Bloch N, Daddacha W, Polsky SB, Schultz ML, Kim B, Landau NR (2012). The Vpx lentiviral accessory protein targets SAMHD1 for degradation in the nucleus. J Virol.

[R24] Hrecka K, Hao C, Gierszewska M, Swanson SK, Kesik-Brodacka M, Srivastava S, Florens L, Washburn MP, Skowronski J (2011). Vpx relieves inhibition of HIV-1 infection of macrophages mediated by the SAMHD1 protein. Nature.

[R25] Kim B, Nguyen LA, Daddacha W, Hollenbaugh JA (2012). Tight interplay among SAMHD1 protein level, cellular dNTP levels, and HIV-1 proviral DNA synthesis kinetics in human primary monocyte-derived macrophages. J Biol Chem.

[R26] Laguette N, Sobhian B, Casartelli N, Ringeard M, Chable-Bessia C, Ségéral E, Yatim A, Emiliani S, Schwartz O, Benkirane M (2011). SAMHD1 is the dendritic- and myeloid-cell-specific HIV-1 restriction factor counteracted by Vpx. Nature.

[R27] Lahouassa H, Daddacha W, Hofmann H, Ayinde D, Logue EC, Dragin L, Bloch N, Maudet C, Bertrand M, Gramberg T (2012). SAMHD1 restricts the replication of human immunodeficiency virus type 1 by depleting the intracellular pool of deoxynucleoside triphosphates. Nat Immunol.

[R28] Lee-Kirsch MA, Chowdhury D, Harvey S, Gong M, Senenko L, Engel K, Pfeiffer C, Hollis T, Gahr M, Perrino FW (2007a). A mutation in TREX1 that impairs susceptibility to granzyme A-mediated cell death underlies familial chilblain lupus. J Mol Med.

[R29] Lee-Kirsch MA, Gong M, Chowdhury D, Senenko L, Engel K, Lee YA, de Silva U, Bailey SL, Witte T, Vyse TJ (2007b). Mutations in the gene encoding the 3^′^-5^′^ DNA exonuclease TREX1 are associated with systemic lupus erythematosus. Nat Genet.

[R30] Li H, Durbin R (2009). Fast and accurate short read alignment with Burrows-Wheeler transform. Bioinformatics.

[R31] Lienenklaus S, Cornitescu M, Zietara N, Lyszkiewicz M, Gekara N, Jablonska J, Edenhofer F, Rajewsky K, Bruder D, Hafner M (2009). Novel reporter mouse reveals constitutive and inflammatory expression of IFN-beta in vivo. J Immunol.

[R32] Morita M, Stamp G, Robins P, Dulic A, Rosewell I, Hrivnak G, Daly G, Lindahl T, Barnes DE (2004). Gene-targeted mice lacking the Trex1 (DNase III) 3^′^—>5^′^ DNA exonuclease develop inflammatory myocarditis. Mol Cell Biol.

[R33] Pascual V, Farkas L, Banchereau J (2006). Systemic lupus erythematosus: all roads lead to type I interferons. Curr Opin Immunol.

[R34] Phuphuakrat A, Kraiwong R, Boonarkart C, Lauhakirti D, Lee TH, Auewarakul P (2008). Double-stranded RNA adenosine deaminases enhance expression of human immunodeficiency virus type 1 proteins. J Virol.

[R35] Powell RD, Holland PJ, Hollis T, Perrino FW (2011). Aicardi-Goutieres syndrome gene and HIV-1 restriction factor SAMHD1 is a dGTP-regulated deoxynucleotide triphosphohydrolase. J Biol Chem.

[R36] Ramantani G, Kohlhase J, Hertzberg C, Innes AM, Engel K, Hunger S, Borozdin W, Mah JK, Ungerath K, Walkenhorst H (2010). Expanding the phenotypic spectrum of lupus erythematosus in Aicardi-Goutières syndrome. Arthritis Rheum.

[R37] Ramantani G, Häusler M, Niggemann P, Wessling B, Guttmann H, Mull M, Tenbrock K, Lee-Kirsch MA (2011). Aicardi-Goutières syndrome and systemic lupus erythematosus (SLE) in a 12-year-old boy with SAMHD1 mutations. J Child Neurol.

[R38] Rampazzo C, Miazzi C, Franzolin E, Pontarin G, Ferraro P, Frangini M, Reichard P, Bianchi V (2010). Regulation by degradation, a cellular defense against deoxyribonucleotide pool imbalances. Mutat Res.

[R39] Ravenscroft JC, Suri M, Rice GI, Szynkiewicz M, Crow YJ (2011). Autosomal dominant inheritance of a heterozygous mutation in SAMHD1 causing familial chilblain lupus. Am J Med Genet A.

[R40] Reijns MA, Rabe B, Rigby RE, Mill P, Astell KR, Lettice LA, Boyle S, Leitch A, Keighren M, Kilanowski F (2012). Enzymatic removal of ribonucleotides from DNA is essential for mammalian genome integrity and development. Cell.

[R41] Rice G, Newman WG, Dean J, Patrick T, Parmar R, Flintoff K, Robins P, Harvey S, Hollis T, O’Hara A (2007a). Heterozygous mutations in TREX1 cause familial chilblain lupus and dominant Aicardi-Goutieres syndrome. Am J Hum Genet.

[R42] Rice G, Patrick T, Parmar R, Taylor CF, Aeby A, Aicardi J, Artuch R, Montalto SA, Bacino CA, Barroso B (2007b). Clinical and molecular phenotype of Aicardi-Goutieres syndrome. Am J Hum Genet.

[R43] Rice GI, Bond J, Asipu A, Brunette RL, Manfield IW, Carr IM, Fuller JC, Jackson RM, Lamb T, Briggs TA (2009). Mutations involved in Aicardi-Goutières syndrome implicate SAMHD1 as regulator of the innate immune response. Nat Genet.

[R44] Rice GI, Kasher PR, Forte GM, Mannion NM, Greenwood SM, Szynkiewicz M, Dickerson JE, Bhaskar SS, Zampini M, Briggs TA (2012). Mutations in ADAR1 cause Aicardi-Goutières syndrome associated with a type I interferon signature. Nat Genet.

[R45] Rönnblom L, Alm GV, Eloranta ML (2011). The type I interferon system in the development of lupus. Semin Immunol.

[R46] Skarnes WC, Rosen B, West AP, Koutsourakis M, Bushell W, Iyer V, Mujica AO, Thomas M, Harrow J, Cox T (2011). A conditional knockout resource for the genome-wide study of mouse gene function. Nature.

[R47] Stetson DB, Ko JS, Heidmann T, Medzhitov R (2008). Trex1 prevents cell-intrinsic initiation of autoimmunity. Cell.

[R48] Tervo HM, Goffinet C, Keppler OT (2008). Mouse T-cells restrict replication of human immunodeficiency virus at the level of integration. Retrovirology.

[R49] Tüngler V, Staroske W, Kind B, Dobrick M, Kretschmer S, Schmidt F, Krug C, Lorenz M, Chara O, Schwille P, Lee-Kirsch MA (2013). Single-stranded nucleic acids promote SAMHD1 complex formation. J Mol Med.

[R50] White TE, Brandariz-Nuñez A, Valle-Casuso JC, Amie S, Nguyen L, Kim B, Brojatsch J, Diaz-Griffero F (2013a). Contribution of SAM and HD domains to retroviral restriction mediated by human SAMHD1. Virology.

[R51] White TE, Brandariz-Nuñez A, Valle-Casuso JC, Amie S, Nguyen LA, Kim B, Tuzova M, Diaz-Griffero F (2013b). The retroviral restriction ability of SAMHD1, but not its deoxynucleotide triphosphohydrolase activity, is regulated by phosphorylation. Cell Host Microbe.

[R52] Yan N, Regalado-Magdos AD, Stiggelbout B, Lee-Kirsch MA, Lieberman J (2010). The cytosolic exonuclease TREX1 inhibits the innate immune response to human immunodeficiency virus type 1. Nat Immunol.

[R53] Yang YG, Lindahl T, Barnes DE (2007). Trex1 exonuclease degrades ssDNA to prevent chronic checkpoint activation and autoimmune disease. Cell.

