# Supplementary material for: Mouse SAMHD1 Has Antiretroviral Activity and Suppresses a Spontaneous Cell-Intrinsic Antiviral Response
Source: Cell Rep. Author manuscript; Available in PMC 2016 Mar 25. (PMC4807655; doi:10.1016/j.celrep.2013.07.037)
Supplement: Extended Experimental Procedures and Supplemental Figures [file NIHMS766882-supplement-supplement_1.pdf]

Supplemental Information

Cell PRESS Open ACCESS

EXTENDED EXPERIMENTAL PROCEDURES

Mice

SAMHD1-deficient mice were generated by injection of targeted ES cell clone JM8A3.N1 (EPD0424_3_A06; http://www.knockoutmouse.org) into C57BL/6 blastocysts. Mutant offspring from chimeric mice was intercrossed to generate homozygous *SAMHD1 "knock out first" (KOF)* mice. The gene trap cassette of the *KOF* allele was deleted in vivo by breeding to Flpe-deleter mice to yield $SAMHD1^{FLOX}$ mice in which *exon2* was flanked by *loxP* sites. Cre-mediated deletion of *exon2* by crossing to a PGK-Cre strain resulted in $SAMHD1^{\Delta}$ mice. Flpe-deleter and PGK-Cre mice were on the C57BL/6 background. Genotyping of all mice was done by Southern Blot (primers for amplification of the probe were: probe3-1-for 5′CCG-AGA-CTG-ACT-CAG-CTG-CTG-GAT-ACA-TTC3′, probe3-1-rev 5′ATA-TGA-GCG-TCT-GAA-GGG-CGT-GGT-CCT-TGA3′) and PCR (SAM-Type-1 5′CAG-TCC-TGG-TGC-ACA-CAT-AC3′, SAM-Type-2 5′AAG-ACC-TAC-AAA-GAG-GGC-GG3′, SAM-Type-3 5′GGG-TGT-ACA-GAG-GTT-AGA-TGC3′, SAM-Type-KOF 5′TAG-GGT-ACC-CCA-GGC-TTC-AC3′).

Western Blot

10^6 splenocytes were lysed in RIPA buffer (50mM Tris-HCl, pH7.4, 150mM NaCl, 1% NP-40, 0.5% sodium deoxycholate, 0.1% SDS) for 30 min on ice. Lysates were cleared by centrifugation (16,000 g, 5 min) and the supernatant was boiled for 5 min in 5x protein loading buffer (Fermentas) containing 100mM DTT. Proteins were separated on a 10% denaturing acrylamide gel and subsequently blotted onto a nitrocellulose membrane (Amersham Hybond-ECL, GE Healthcare). After blocking for one hour (tris-buffered saline, 0.05% Tween 20,5% bovine serum albumin), the SAMHD1-specific antibody (3F5, Abcam, 1:500) was added over night at 4°C with agitation. Following three washes, the membrane was incubated for one hour with a polyclonal rabbit-anti-mouse IgG-HRP antibody conjugate (final dilution 1:1000, Dako). Signals were detected on an LAS-3000 image analyzer (Fujifilm Europe) using the Amersham ECL Plus Western Blotting Detection System (GE Healthcare).

Virus Preparation

Reporter virus was produced in 293T cells co-transfected with *env*-deficient reporter virus plasmids and pVSV-G vesicular stomatitis virus glycoprotein expression plasmid using calcium phosphate co-precipitation. HIV-1-eGFP (VSV-G/NL43-CMVGFP) was generated by co-transfection of pNL43-E-CMVGFP and pVSV-G at a mass ration of 4:1 as described previously (Hofmann et al., 2012). HIV-1-GFP (VSV-G/HR.CMVGFP) used for in vivo infections was generated by co-transfection of the lentiviral vector pHR.CMVGFP, the packaging plasmid pCMVΔR8.91, and pVSV-G at a ratio of 2:2:1 (Tervo et al., 2008). Lentiviral particles encoding codon-optimized murine SAMHD1 or control particles were generated by co-transfection of p6NST56-mucoSAMHD1-IRES-YFP or empty p6NST56-IRES-YFP vector, pCMVdeltaR8.91, and pVSV-G. For all transfections, the culture medium was replaced after 6h. Supernatants were harvested 48h posttransfection, passed through 0.4 μm-pore size filters, aliquotted and frozen at −80°C. HIV-1-CMVGFP particles for in vivo infections were additionally purified by pelleting through 20% sucrose at 32,000 g for 2h at 4°C and resuspended in PBS. All GFP reporter viruses were titered on 293T cells and infectivity was determined 72h postinfection by flow cytometry. VLP were normalized for SIV Gag content (p27) measured by ELISA (Innogenetics).

In Vitro Differentiation of BMDCs

Bone marrow cells were differentiated in vitro for 7 days in RPMI 1640 Medium (Biochrom) supplemented with 10% heat-inactivated fetal calf serum (Biochrom), 10ng/ml GM-CSF (PeproTech), 100U/ml Penicillin, 100μg/ml Streptomycin (Biochrom), 1mM sodium pyruvate (Biochrom) and 2mM L-Alanyl L-glutamine (Biochrom).

Friend Virus Infection

For infection of mice with Friend virus (FV), the B-tropic, polycythemia-inducing FV-complex used in all experiments was from uncloned virus stocks obtained from 10% spleen cell homogenates as described (Hasenkrug et al., 1998). The progression of disease was monitored by spleen weights and virus assays as indicated. Mice were injected intra-venously with 0.5ml PBS containing 20,000 spleen focus-forming units (SFFU) of the FV complex which was not contaminated by Lactate dehydrogenase virus (LDV). For the analysis of viral loads, infectious centers from spleens and bone marrow were detected by 10-fold dilutions of single cell suspensions on Mus dunnis cells. Cultures were incubated for 5 days, fixed with ethanol, stained with F-MuLV envelope-specific monoclonal antibody 720 (Dittmer et al., 1998) and developed with peroxidase-conjugated goat anti-mouse antibody and aminoethylcarbazol to detect foci.

Illumina-Based Transcriptome Analysis

Cell-lysates were cleaned with LysateClear Columns (Miltenyi Biotec), heated for 5 min to 65°C and placed on ice. mRNA was enriched using 15μl Sera-Mag Oligo(dT) beads (Thermo Scientific) according to the manufacturer's instructions and eluted in 50μl 10mM Tris-HCl. Samples were digested with 1U Turbo DNase (Ambion) and purified with Agencourt RNAclean XP beads (Beckman Coulter). mRNA (18 μl) was chemically fragmented with NEBNext Magnesium RNA Fragmentation Module (NEB), purified with RNAclean XP beads and eluted in 13.5 μl nuclease free water. First strand cDNA synthesis was performed using 0.15μg/μl Random Primers (NEB), 1x First Strand Synthesis Reaction Buffer (NEB), 10U/μl Superscript II (Invitrogen) with an initial hybridization at 65°C

for 5 min followed by incubation at 25°C for 10 min, 42°C for 50 min and 70°C for 15 min. After purification with Agencourt AMPure XP beads (Beckman Coulter) according to the manufacturer's instructions, second strand synthesis was performed using a modified version of the Second Strand Synthesis module (NEB) in which the 2nd strand synthesis buffer was replaced with an NTP-free buffer supplemented with 2.5mM each dUTP, dATP, dGTP and dCTP. Incubation for 2.5h at 16°C was followed by Ampure XP beads purification as described above. End-Repair was done with the NEBnext End Repair Module (NEB) according to the manufacturer's instructions followed by XP bead purification and A-Tailing according to NEB instructions using the NEBnext dA-Tailing Module. After purification, adaptors were ligated (Adaptor-Oligo 1: 5′ACA-CTC-TTT-CCC-TAC-ACG-ACG-CTC-TTC-CGA-TCT3′, Adaptor-Oligo 2: 5′P-GAT-CGG-AAG-AGC-ACA-CGT-CTG-AAC-TCC-AGT-CAC3′) using 1x NEBnext Quick Ligation Buffer (NEB), 0.3μM DNA Adaptors, 1μl Quick T4 DNA Ligase (NEB) in 50μl total volume. XP bead purification was followed by dUTP cleavage by digest with 1U USER enzyme mix (NEB) per sample and direct enrichment of the library constructs using the PCR Enrich Adaptor Ligated cDNA Library module (NEB) with indexed primers. After XP bead purification, libraries were quantified using Qubit dsDNA HS Assay Kit (Invitrogen). Sequencing was done on the Illumina® HiSeq2000, on a 75 bp single read run on the same flow cell, resulting in 12-25 million reads per sample. A splice junction library with a length of 120 nucleotides (60+60) per splice junction was created with RSEQTools (Habegger et al., 2011). Alignment of the reads to the mm9 transcriptome was performed with the parallel version of BWA (Li and Durbin, 2009), pBWA (http://pbwa.sourceforge.net/), resulting in a mappability range 75.6% - 77.2% (for the uniquely mapped reads, meaning reads that map uniquely to a position and not randomly). A table of counts per gene was created based on the overlap of the uniquely mapped reads with the Ensembl Genes annotation for mm9 (v. 61, Feb. 2011) using BEDtools. The raw counts (absolute number of reads) were normalized with the DESeq R package (v.1.6.1) (Anders and Huber, 2010) and sample to sample correlation (Pearson's correlation coefficient) was computed based on the normalized counts, resulting in 100% between biological replicates and 98%–99% between wild-type and mutant. After normalization, testing for differential expression between wild-type and SAMHD1-deficient mice was performed with DESeq; the count data were fitted to the negative binomial distribution and the p-values for the statistical significance of the fold change were adjusted for multiple testing with the Benjamini-Hochberg correction for controlling the false discovery rate. The false discovery rates were 5% (padj $\leq$ 0.5) and 10% (padj $\leq$ 0.10) for the first and the second experiment, respectively.

Quantitative RT-PCR

Total RNA was isolated using RNeasy Mini Kit (QIAGEN) following the manufacturer's instructions. Reverse transcription of 250–500ng total RNA was performed using the RevertAid™ H Minus First Strand cDNA Synthesis Kit (Fermentas) and oligo(dT) primer. 1μl cDNA served as template for the quantitative RT PCR (Maxima® SYBR Green/ROX qPCR Master Mix (Thermo Scientific), 2pmol gene-specific primers) with the following cycling conditions on a Mx3005P QPCR system (Agilent Technologies): 10 min 95°C, 40 cycles of 95°C for 30 s, 58°C for 30 s and 72°C for 15 s. Transcript levels were analyzed with the MxPro QPCR Software (Agilent Technologies). The following RT PCR primers were used: SAMHD1-RT1 5′GGG-AAC-CGG-AGG-ACG-TGT-GC3′, SAMHD1-RT2 5′TCT-TCC-AGA-CGA-TCC-TCA-TC3′, SAMHD1-RT6R 5′GCG-AGC-CCG-TGG-GAT-AAA-CCT-TCC3′, Ifi44-For 5′GGC-ACA-TCT-TAA-AGG-GCC-ACA-CTC3′, Ifi44-Rev 5′CTG-TCC-TTC-AGC-AGT-GGG-TCA-TG3′, Pydc4-For 5′CAT-TCC-AGA-ACT-TGC-AGC-TCG-TG3′, Pydc4-Rev 5′GTA-AGT-GGA-GGA-GGG-CTG-GAT-TC3′, Oasl1-For 5′CGT-TGT-GCC-CGC-CTA-CAG-AGC-C3′, Oasl1-Rev 5′GCT-GCA-GCT-CGC-TGA-AGG-ATG-G3′, Rsad2-For 5′CAA-GCG-AGG-ACT-GCT-TCT-GCT-C3′, Rsad2-Rev 5′GCA-GAA-TCT-CAC-AAG-CTT-GCC-C3′, Usp18-For 5′CAC-AAC-ATC-GGA-CAG-ACG-TGT-TGC3′, Usp18-Rev 5′CTT-CCT-CTC-TTC-TGC-ACT-CCG-AG3′, GAPDH-For 5′AAG-GGG-CGG-AGA-TGA-TGA-C3′, GAPDH-Rev 5′GGT-GCT-GAG-TAT-GTC-GTG-GAG3′, TBP-For 5′TCT-ACC-GTG-AAT-CTT-GGC-TGT-AAA3′, TBP-Rev 5′TTC-TCA-TGA-TGA-CTG-CAG-CAA-A3′. Transcript levels were normalized to GAPDH or TBP. All samples were run in technical triplicates.

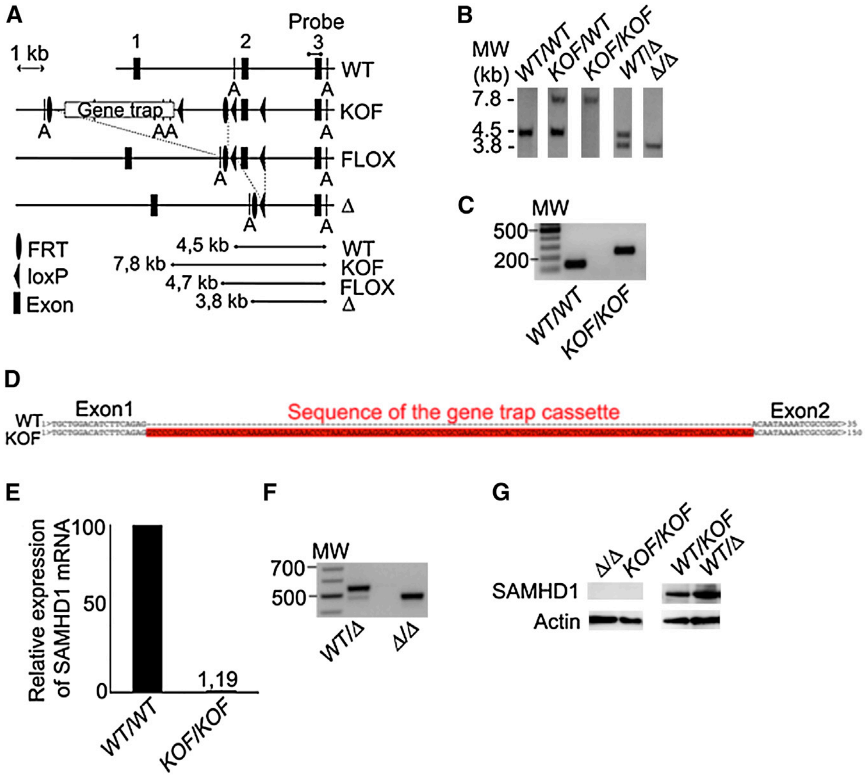

Figure S1. Targeted Inactivation of the *SAMHD1* Gene, Related to Results

(A) Targeted insertion of a gene trap cassette into *intron1/2* yielded *SAMHD1*^{KOF} mice (International Knockout Mouse Consortium) in which splicing of the cassette to *exon1* results in a sterile transcript with premature stops. Flpe- and Cre-mediated in vivo-deletion of the gene trap cassette and *loxP*-flanked *exon2*, respectively, resulted in *SAMHD1*$^{\Delta}$ mice. Deletion of *exon2* introduces a frame shift. A, ApaLI; probe, position of probe for Southern blot.

(B) Southern blot analysis of ApaLI-digested tail DNA of the indicated genotypes.

(C) Amplification of *SAMHD1* mRNA from *SAMHD1*$^{KOF/KOF}$ splenocytes using *exon1*- and *exon2*-specific primers yielded a single distinct product that was 115 bp longer than the product expected for wild-type mRNA, which was undetectable.

(D) Sequencing of this product demonstrated that the gene trap cassette was spliced to *exon1* as expected. A cryptic splice donor site 115 bp downstream of the gene trap splice acceptor was used to splice to *exon2* resulting in a frame shift and premature stops.

(E) Quantification of transcript levels by qRT-PCR using *exon1*- and *exon2*-specific primers. Compared to the *SAMHD1* transcript in wild-type cells, the mutant mRNA of *SAMHD1*$^{KOF/KOF}$ cells was reduced to 1.19% (n = 3 each genotype). Means ± SD are displayed.

(F) Amplification of *SAMHD1* transcripts from *SAMHD1*$^{\Delta/\Delta}$ macrophages using *exon1* and *exon6*-specific primers yielded a single product corresponding to the expected splicing of *exon1* to *exon3*, resulting in a frame shift and premature stops. Splicing of *exon1* to *exon3* was verified by sequencing of the RT-PCR product (not shown). Nonsense-mediated RNA decay most likely caused the reduced levels of mutant compared to wild-type transcripts in heterozygous cells confirming the reduction of mutant *SAMHD1* transcripts to 36% of control observed in the NGS mRNA sequencing analysis (GEO accession number GSE37236).

(G) Western blot analysis of *SAMHD1*$^{KOF/KOF}$ and *SAMHD1*$^{\Delta/\Delta}$ splenocytes demonstrates absence of SAMHD1 protein. All panels in (B) and (G) each originate from the same blot but were rearranged for presentation.

Cell PRESS
Open ACCESS

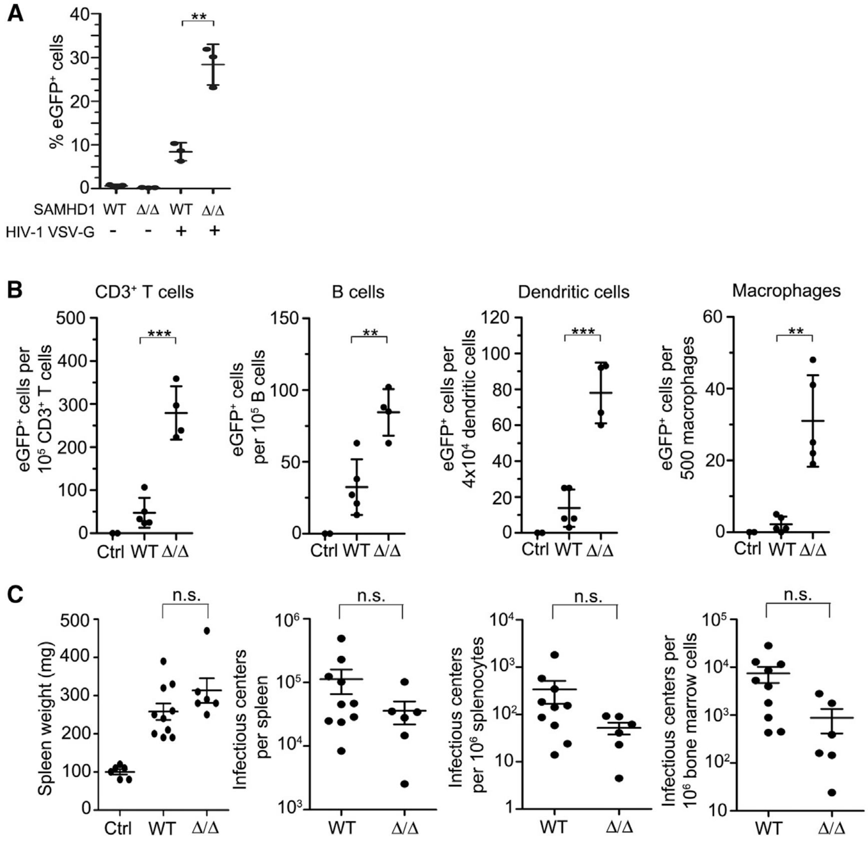

Figure S2. Murine SAMHD1 Restricts HIV-1 but Does Not Seem to Control Replication of Friend Leukemia Virus In Vivo, Related to Figure 2

(A) In vitro infection of *SAMHD1*$^{\Delta/\Delta}$ and *SAMHD*$^{WT/WT}$ BMDCs (n = 3 both groups) with eGFP-containing HIV-1-VSV-G (MOI = 1). Flow cytometric quantification of eGFP$^+$ BMDCs three days after infection is shown. As expected, mock-infected cultures displayed only marginal fractions of fluorescent cells. *SAMHD*$^{WT/WT}$ BMDC cultures contained about 8% eGFP$^+$ cells. This fraction was increased about three- to four-fold in cultures of *SAMHD1*$^{\Delta/\Delta}$ cells. Means ± SD are displayed.

(B) Differential analysis of individual splenic cell populations from the HIV reporter virus infection of five *SAMHD1*$^{\Delta/\Delta}$ and five *SAMHD1*$^{WT/WT}$ mice shown in Figure 2C. Absolute numbers of live eGFP$^+$ cells in the indicated populations are shown. Means ± SD are displayed.

(C) *SAMHD1*$^{\Delta/\Delta}$ (n = 6) and control (n = 10) mice were infected with Friend retrovirus (FV). No difference was detected in spleen weight or viral load in spleen and bone marrow ten days post infection. Means ± SEM are displayed.

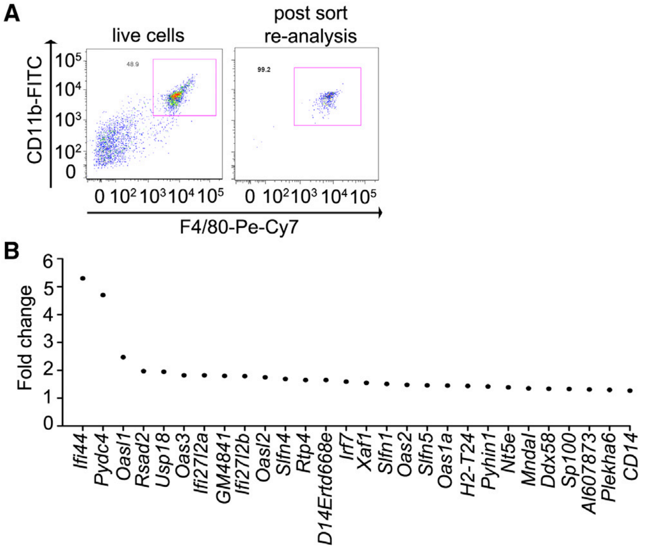

Figure S3. Spontaneous Induction of Type I IFN-Inducible Genes in *SAMHD1*$^{\Delta/\Delta}$ Mice, Related to Figure 3

(A) Gating of CD11b^{+}F4/80^{+} macrophages from peritoneal lavage fluid for flow cytometric sorting and representative example of a post-sort re-analysis demonstrating purity of the sorted macrophages above 99%.

(B) mRNA of FACS-sorted peritoneal macrophages from nine *SAMHD1*$^{\Delta/\Delta}$ and nine control mice was analyzed by Illumina®-based transcriptome sequencing and confirmed the results of the first transcriptome analysis (Figure 3). Sorted macrophages from three animals were combined to yield three pools per group. 28 genes significantly (padj $\leq$ 0.1) upregulated in mutant versus control samples were identified. As in the first experiment, most of these represented type I IFN-inducible genes (see also Table S1).

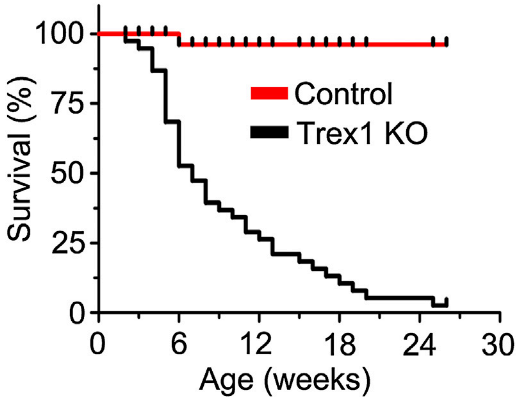

Figure S4. Survival of TREX1-Deficient Mice, Related to Figure 4

Under the conditions of our animal facility, *Trex1*$^{KO/KO}$ mice (KO, n = 38) died within few months as expected (Control, *Trex1*$^{WT/-}$ or *Trex1*$^{WT/WT}$, n = 38, log-rank test, p < 0,001), with a median survival of seven weeks. Others studies reported median survival of nine (Stetson et al., 2008) or 17 weeks (Beck-Engeser et al., 2011; Morita et al., 2004) for *Trex1*$^{-/-}$ mice.
