# Supplementary material for: Mouse SAMHD1 Has Antiretroviral Activity and Suppresses a Spontaneous Cell-Intrinsic Antiviral Response
Source: Cell Rep. Author manuscript; Available in PMC 2016 Mar 25. (PMC4807655; doi:10.1016/j.celrep.2013.07.037)
Supplement: Table S1 [file NIHMS766882-supplement-Table_S1.pdf]

Table S1. Reports of type I-IFN-dependent induction of genes found up-regulated in SAMHD1-deficient mice. Related to Figure 3 and Figure S3.

gene	publication	www.interferome.org/	GEO accession no. GSE35825[1, *]	GEO accession no. GSE36891[1, **]
Ifi44	Kitamura et al., 1994, Eur J Biochem., 224: 877-883; Hallen et al., 2007, J Interferon Cytokine Res., 27:675–680	+	+	+
Pydc4			+	+
Gm4955	Ludlow et al., 2008, J Cell Biochem., 103(4):1270-82			+
Pydc3				
ENSMUSG00000090158				
Oasl1	Eskildsen et al., 2002, Cell Mol Life Sci., 59: 1212–1222; Eskildsen et al., 2003, Nucleic Acids Res., 31(12): 3166-3173	+	+	+
Adam33			+	
Gm2619				
Ifi205	Asefa et al., 2006, FEBS Lett., 580(5):1205-14	+	+	+
Iigp1	Uthaiah et al., 2003, J Biol Chem., 278(31):29336-43		+	+
BC094916			+	+
Ifit2	Bluyssen et al., 1994, Genomics., 24(1):137-48; Wacher et al., 2007, J Virol., 81(2):860-71	+	+	+
Rsad2	Fitzgerald, 2011, J Interferon Cytokine Res., 31(1):131-5.; Mattijssen et al., 2012, Microbes Infect., 14(5): 419-26	+	+	+
Ifit3	Wacher et al., 2007, J Virol., 81(2):860-71; Schmeisser et al., 2010, J Virol., 84(20):10671-80	+		+
BC023105				+
Gm4841				+
Gm7592			+	+
Ifi27l2a	Gjermandsen et al., 2000, Cytokine, 12(3):233-8; Cheriyath et al., 2011, J Interferon Cytokine Res., 31(1)	+	+	+
Ifit1	Bluyssen et al., 1994, Genomics., 24(1):137-48; Wacher et al., 2007, J Virol., 81(2):860-71	+	+	+
Ifi27l2b	Lu et al., 2011, Cell Death Differ., 18(6):925-36		+	
Usp18	Malakhov et al., 2002, J Biol Chem., 277(12): 9976–9981	+	+	+
Ms4a6b			+	+
Tgtp2		+		+
Siglec1	York et al., 2007, Arthritis Rheum., 56(3):1010-20		+	+

Ms4a6c			+	+
Irf7	Sato et al., 1998, FEBS Lett., 441: 106-110; Zimmerer et al., 2007, Cancer Immunol Immunother., 56:1845–1852	+	+	+
C130026I21Rik		+	+	+
Oasl2	Eskildsen et al., 2002,Cell Mol Life Sci., 59: 1212–1222; Eskildsen et al., 2003, Nucleic Acids Res., 31(12): 3166-3173		+	+
Gm16026				
Oas3	Eskildsen et al., 2002,Cell Mol Life Sci., 59: 1212–1222	+	+	+
Isg15	Reich et al., 1987, Proc Natl Acad Sci U S A., 84(18):6394-8; Darnell et al., 1994, Science., 264(5164):1415-21	+	+	+
Mx1	Chang et al.,1991, Arch Virol., 117(1-2):1-15; Holzinger et al.,2007, J Virol. 2007, 81(14):7776-85		+	+
A530032D15Rik				+
Pyhin1	Ludlow et al., 2005, Exp Cell Res., 308: 1– 17; Schattgen et al., 2011,Immunol Rev., 243: 109–118		+	+
ENSMUSG00000079459				

[1] accessed on 11/01/2013

* studies with IFN-α treatment

** studies poly(IC) treatment
